# Humeral condylar fractures and fissures in the French bulldog

**DOI:** 10.1111/vsu.13907

**Published:** 2022-10-12

**Authors:** Oliver J. Anderson, Sorrel J. Langley‐Hobbs, Kevin J. Parsons

**Affiliations:** ^1^ Langford Small Animal Hospital University of Bristol Bristol UK

## Abstract

**Objective:**

To report the configuration, risk factors, fixation methods and complication rates after repair of humeral condylar fractures (HCF) in French bulldogs, and report the presence of humeral intracondylar fissures (HIF) in this population as a possible predisposing factor.

**Study design:**

Retrospective clinical cohort study.

**Sample population:**

Forty‐four elbows.

**Methods:**

The medical records of dogs referred between January 2012 and December 2021 were searched for French bulldogs presenting with HCF. Signalment, fracture configuration, stabilization method and complication occurrence were obtained. Postoperative radiographs were assessed for implant positioning, and computed tomography (CT) scans were assessed for the presence and size of HIF in the contralateral elbow.

**Results:**

Lateral humeral condylar fractures represented 28/44 (63.6%) of HCF in French bulldogs. Repair with a transcondylar screw (TCS) and Kirschner‐wire(s) (K‐wire) were 7.62 times more likely to result in a major complication (95% CI: 1.43, 21.89; *p* = .01) compared to other methods. All incidences (7/7) of TCS migration were within the TCS + K‐wire group. A HIF was identified in 18/31 (58.1%) dogs. Older animals were not significantly less likely to have a HIF than younger animals (*p* = .129).

**Conclusions:**

Fracture stabilization with a TCS and K‐wire(s) was associated with an increased risk of major complications and migration of the TCS. A HIF was present in the contralateral elbow of over half of the French bulldogs where CT was available.

**Clinical significance:**

A HIF may be a predisposing factor of HCF in French bulldogs. Alternative methods of stabilization to a TCS and K‐wire(s) should be used to reduce complication risk.

**Abbreviations**
BCF: bicondylar fractures
CW: condylar width
HCF: humeral condylar fractures
HIF: humeral intracondylar fissures
LHCF: lateral humeral condylar fractures
MHCF: medial humeral condylar fractures
SD: screw core diameter
TCS: transcondylar screw
TSA: transcondylar screw angle

## 1 INTRODUCTION

The majority of distal humeral fractures in dogs involve the humeral condyle.^1^ A number of breeds have been found to be predisposed to humeral condylar fractures (HCF) including spaniels,^1^, ^2^, ^3^, ^4^ Yorkshire terriers,^5^, ^6^ Miniature schnauzers, Gordon setters,^6^ Cavalier King Charles spaniels^7^ and the French bulldog.^6^, ^8^ HCF are most commonly diagnosed in young, skeletally immature dogs, with the most common age being around 4 months when ossification of the humeral condyle is not yet complete,^1^, ^5^, ^7^, ^9^ or in older dogs with an underlying humeral intracondylar fissures (HIF).^10^ HCF can be categorized as lateral condylar, medial condylar and bicondylar fractures (more commonly described as “Y” or “T” fractures).^2^ Overall, lateral humeral condylar fractures (LHCF) are most common, accounting for approximately 56%–67% of all HCF. Medial humeral condylar fractures (MHCF) account for 4%–16% and bicondylar fractures (BCF) for 33%–35%.^1^, ^3^, ^5^, ^6^, ^7^

Two possible factors are postulated to explain why LHCF are more common than BCF and MHCF. First, the lateral epicondylar region is smaller and therefore weaker than the medial region. Second, the radius articulates predominantly with the lateral part of the condyle and therefore forces from sudden impacts are predominantly directed laterally.^5^, ^9^, ^11^ Fractures are commonly sustained following falls or jumps.^5^, ^6^

Humeral condylar fractures are commonly diagnosed without evidence of a history of trauma. In these cases, a humeral intracondylar fissure (HIF) is probably the predisposing factor. There are two principal theories as to the formation of HIFs. A HIF may represent a failure of the medial and lateral secondary centers of ossification of the humeral condyle to fuse during skeletal development (hence HIF previously being known as incomplete ossification of the humeral condyle or IOHC).^12^ In certain populations of dogs, such as skeletally mature animals, a HIF may also represent a stress fracture of the humeral condyle.^13^, ^14^ A HIF could therefore be a single presentation for two separate etiologies.

Humeral condylar fractures are typically stabilized using internal fixation with the aim of achieving compression of the fracture fragments, anatomical reconstruction and reduction of the articular surface. The most common method employed involves the use of a transcondylar screw (TCS) along with antirotational pins or a plate, but other techniques have been described.^2^, ^9^, ^15^, ^16^, ^17^, ^18^, ^19^, ^20^, ^21^, ^22^, ^23^, ^24^ Fixation is not without risk, and complications associated with surgery can often affect the overall outcome. Complications have been reported to occur in between 15%–41.5% of cases.^1^, ^5^, ^15^, ^21^, ^24^, ^25^, ^26^ Fixation of LHCF using a TCS alongside a lateral epicondylar plate as stabilization resulted in reduced postoperative complications compared to other methods.^8^, ^24^ Reported complications include persistent lameness, reduced range of motion, elbow arthrosis, nonunion, fixation failure, seroma formation and infection.^1^, ^3^, ^4^, ^7^, ^17^, ^24^, ^26^ Other negative outcomes associated with HCF can include long‐term pain and lameness, with these signs reported in 28%–57% of dogs post‐surgery.^1^, ^3^, ^7^ With early reduction and stabilization, the prognosis for both medial and lateral condylar fractures is generally good. The severity of osteoarthritis following HCF can be minimized by accurate reduction.^27^ The prognosis for BCF is more guarded;^2^, ^7^ however, a recent study found bilateral fixation of BCF led to a good or excellent outcome in 27/30 fractures.^28^

The popularity of the French bulldog has seen an unprecedented rise in the United Kingdom over the last 10 years, with a 1417% increase in Kennel Club registrations from 2011–2020.^29^ A marked increase in the number of French bulldogs presenting with HCF has been observed at the authors’ institution. It is unclear if there is an underlying etiology for this, or if it is related to their increased popularity. A predisposition in spaniel breeds to HCF has been linked to HIF,^10^ but the pathogenesis in French bulldogs remains unknown. Although documented as a predisposed breed, there are limited studies looking into HCF in French bulldogs alone.^8^, ^30^, ^31^, ^32^ French bulldogs have a different predisposition of fracture configuration, with a higher rate of MHCF compared to other dogs.^8^ The incidence of HIF within the contralateral limb of French bulldogs presenting with HCF has also been previously reported, with no dogs having evidence of a fissure.^8^ This is in contrast to the experience of cases seen at the authors’ institution, where contralateral HIF has been identified on CT scans in French bulldogs presenting with unilateral HCF. A single case series of nine dogs investigated whether HIF in French bulldogs is a contributing factor to HCF, with 5/9 dogs having suspicion of a predisposing HIF.^31^ To the authors’ knowledge, although extensively studied in spaniels, there is no study looking at fracture type, method of fixation, and complication rates for HCF in the French bulldog as an individual breed.

The aim of this study was to retrospectively review cases of HCF in French bulldogs presenting to a single referral center in the United Kingdom. It was our perception that a fracture of the lateral aspect of the humeral condyle is the most common fracture configuration in French bulldogs and that the incidence of a humeral condylar fissure in the contralateral elbow is higher than previously reported. We also perceived that the complication rate was higher in dogs whose fractures were stabilized with a TCS and antirotational K‐wire. Our hypotheses were therefore (1) LHCF are more common than MHCF and BCF in French bulldogs, (2) The complication rate is higher for HCF stabilized with a TCS and antirotational K‐wire(s) (3) The presence of a HIF in the contralateral elbow is due to delayed ossification (IOHC) and therefore the incidence decreases with age.

## 2 MATERIALS AND METHODS

### 2.1 Study population

Medical records (January 2012–December 2021) from a single referral hospital were retrieved for all French bulldogs that presented for surgical stabilization of HCF. Dogs that were not treated surgically, or where postoperative data was not available, were excluded from the study. The data retrieved included age, weight, sex, fracture type (LHCF, MHCF, BCF), etiology of fracture, stabilization method used to stabilize the fracture and whether evidence of a HIF existed in the contralateral limb on CT (if available).

### 2.2 Clinical data collection

Postoperative complications were recorded, and details noted. Complications were defined as any undesirable outcome related to the surgery as previously detailed by Cook et al.^33^ Postoperative complications specifically recorded included the presence of seroma formation and radiographic evidence of screw loosening. Due to the small number of fractures, the minor and no complication groups were combined for statistical analysis. This resulted in a major complication group (medical or surgical intervention) and a nonintervention group that included minor complications.

### 2.3 Diagnostic imaging data collection

**FIGURE 1:**
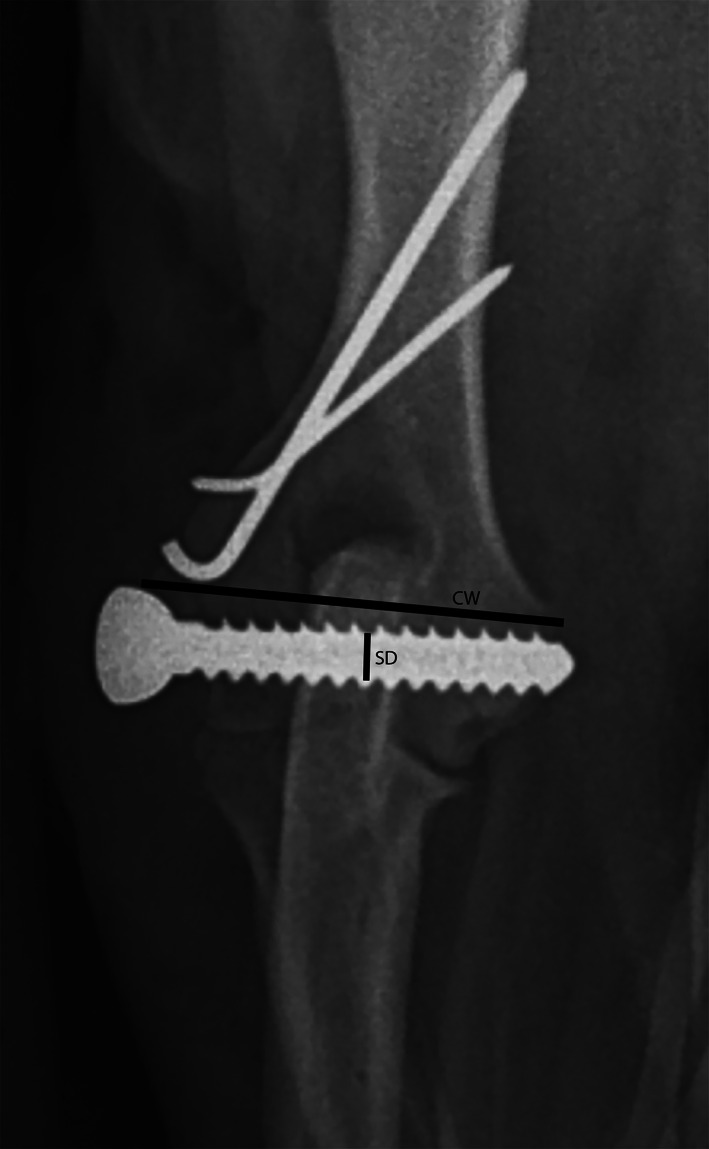
The measurements taken for the condylar width (CW), screw core diameter (SD) and transcondylar screw angle (TSA).

For all elbows where postoperative radiographs were available (44/44), the radiographs were assessed as previously described by Morgan et al.^26^ This included screw core diameter (SD); transcondylar screw angulation (TSA) relative to a line between the medial and lateral epicondyles as viewed on a craniocaudal radiograph; condylar width (CW) measured from the lateral epicondyle to the medial epicondyle. All measurements were made directly from the radiographs to standardize the measuring of these variables (Figure 1). Radiographs were standardized for measurement using an orthopedic marker. A ratio termed the CW to SD (CW:SD) was then calculated from the above measurements to normalize the measurements across case sizes. For follow‐up visits, the radiographic reports from a board‐certified radiologist were used to assess for evidence of fracture healing and implant stability.

**FIGURE 2:**
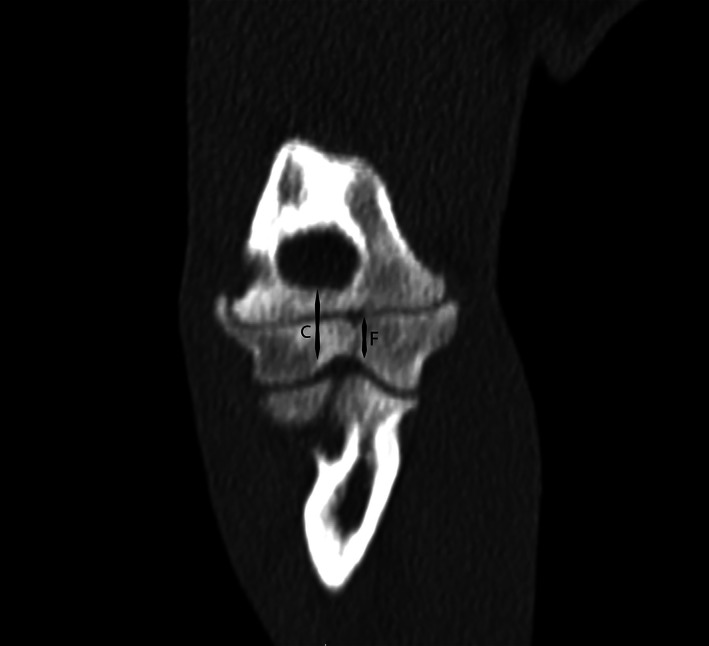
The measurements taken from the contralateral limb CT scan. This shows the measurements taken including the condylar height (C) and the fissure height (F) in order to calculate the percentage fissure length.

**FIGURE 3:**
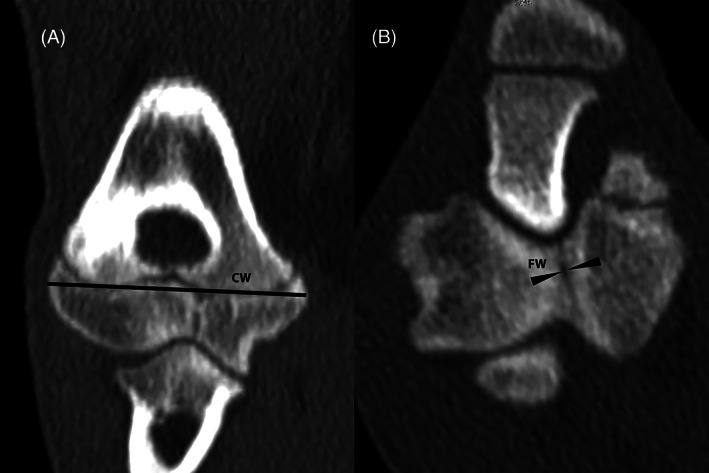
The measurements taken from the contralateral limb CT scan. This includes the condylar width (CW) in the frontal plane (A) and the fissure width (FW) in the transverse plane (B). The CW:FW is then calculated.

For dogs where CT of the contralateral limb was available (31/42), images were reviewed for evidence of a HIF in the contralateral limb. As described by Moores and Moores,^10^ a multiplanar reconstruction of the humeral condyle was created. Because the two‐dimensional size of incomplete fissures cannot be objectively measured in a single plane, a subjective assessment of fissure size was made, relative to the size of the sagittal plane of the humeral condyle at the location of the fissure, to produce a percentage fissure length (Figure 2). The width of the fissure was also measured using the widest part of the fissure in the transverse plane, and expressed as a ratio termed CW to fissure width ratio (CW:FW) (Figure 3).

### 2.4 Statistical analysis

Commercially available statistical software program (IBM SPSS Statistics Version 28) was used to perform all statistical analysis, and graphs were produced using Microsoft Excel. Data was assessed for normality using the Shapiro–Wilk test. Nonparametric tests were used to assess data that were not normally distributed. Descriptive statistics were used to report medians and ranges. Values of *p* < .05 were considered statistically significant for all tests.

Associations between all categorical data were assessed using Fisher's exact test or Chi squared as appropriate. For continuous data including TSA, CW:SD and weight, the independent samples *t*‐test, or Mann–Whitney U test were used depending on normality of distribution. The association between continuous data, such as age and fissure size were assessed using Spearman's correlation coefficient (expressed as *R*, where *R* ranges from −1 to 1).

## 3 RESULTS

A total of 54 French bulldogs presented with HCF during the study period. Twelve dogs were excluded as inadequate follow‐up data was available, leaving a total of 42 dogs. Two dogs suffered bilateral HCF and so each limb was treated as an individual fracture. There was therefore a total of 44 total elbows with HCF. Of the HCF, 28/44 (63.6%) were LHCF, 7/44 (15.9%) MHCF and 9/44 (20.5%) BCF. The right forelimb was affected in 27/44 (61.4%) fractures, and the left in 17/44 (38.6%). There were 23 (52.3%) entire males and 21 (47.7%) entire females included. Median age at time of initial presentation was 17 weeks (range 13–56 weeks). Weight ranged from 3.8 to 15 kg (median 6.4 kg). Thirty‐one out of 44 HCFs (70.5%) occurred after a reported minor trauma, 8/44 (18.2%) had no obvious trauma reported and in 5/44 (11.4%) the cause was unknown with the dog just presenting to the owner with lameness (Table 1).

**TABLE 1:** Summary of humeral condylar fracture etiology

	Cause of fracture	Number of cases
Trauma observed	Fallen from sofa/bed	18
	Dropped by owner	7
	Pounced on by cat	1
	Caught in door	1
	Hit by ball	1
	Owner fallen onto dog	1
No overt inciting trauma	Running in garden	2
	Chasing cat	1
	Playing	5
Unknown/none		5
Total		42

### 3.1 Method of stabilization

Twenty‐four out of 44 (54.5%) fractures were stabilized by placement of a TCS and supracondylar K‐wire(s), 19/44 (43.2%) fractures were stabilized using a TCS plus one or more epicondylar plates, and 1/44 (2.3%) was stabilized using a 3.5 mm TCS alone (herein named as the “other” group). One fracture included in the TCS and plate group also underwent a proximal ulnar osteotomy to allow accurate articular reduction during surgery. Table 2 shows the distribution of fracture stabilization for morphology type.

**TABLE 2:** Summary of the methods of repair for each fracture morphology

Fracture type	Number using method of stabilization (%)			
	TCS + K‐wire(s)	TCS + plate	Other	Total
LHCF	20 (71.4)	8 (28.6)	0 (0)	28 (100)
MHCF	3 (42.9)	3 (42.9)	1 (14.3)	7 (100)
BCF	1 (11.1)	8 (88.9)	0 (0)	9 (100)

Abbreviations: BCF, bicondylar fracture; LHCF, lateral humeral condylar fracture; MHCF, medial humeral condylar fracture; TCS, transcondylar screw.

When the TCS and K‐wire(s) group was investigated further, 14/24 (58.3%) used one K‐wire, 9/24 (37.5%) used two K‐wires and 1/24 (4.2%) used three K‐wires. Where more than one K‐wire was used, 6/10 (60%) used a transcondylar and supracondylar wire. The remaining 4/10 (40%) were placed across the supracondylar fracture line as crossed pins.

### 3.2 Follow‐up and outcome

Follow‐up was obtained at a median time of 6 weeks postoperatively (range 3–60 weeks). Twenty‐six out of 44 (59.1%) elbows experienced no complications, 5/44 (11.4%) had minor complications and 13/44 (29.5%) had major complications. No dogs suffered catastrophic complications. This resulted in an overall complication rate of 40.9%. Of the 13 dogs that experienced a major complication, 10/13 (76.9%) required surgical intervention. Further surgery was recommended in one of the 10 elbows due to implant failure, but this was declined by the owner. Three out of 13 (23.1%) required medical intervention. The mean time of major complication occurrence was 6.5 weeks post surgery (range 1–24 weeks). Individual elbows that experienced major complications are documented in Table 3.

**TABLE 3:** Summary of the individual major complications encountered

Case	Method of stabilization	Major complication encountered	Time of complication postoperatively (weeks)	Time of final recheck (weeks)	Comments
1	TCS and K‐wire(s)	Fracture, TCSM KWM	24	30	Replaced with TCS and plate
2	TCS and K‐wire(s)	Seroma	3	30	Treated with NSAIDS
3	TCS and K‐wire(s)	Seroma TCSM	12	28	Removal of TCS
4	TCS and K‐wire(s)	Seroma TCSM	4	7	TCS replaced. The fissure in the contralateral limb was noted as being possibly larger on CT at 7 weeks than at initial presentation
5	TCS and K‐wire(s)	Seroma KWM	12	22	Removal of K‐wire
6	TCS and K‐wire(s)	Seroma KWM	4	6	Removal of K‐wire
7	TCS and K‐wire(s)	Septic arthritis	1	8	Treated with antibiotics
8	TCS and K‐wire(s)	Broken K‐wire TCSM	4	13	Replaced with TCS and plate
9	TCS and K‐wire(s)	Broken K‐wire TCSM	6	6	Repeat surgery advised, but not pursued by owner
10	TCS and K‐wire(s)	Delayed fracture healing	6	16	Suspected implant loosening so TCS and K‐wire replaced
11	TCS and K‐wires(s)	Seroma TCSM KWM	3	6	Removal of K‐wire
12	TCS and plate	Seroma	4	4	Suspected due to long screw. TCS replaced with shorter screw
13	TCS and plate	Septic arthritis	2	3	Treated with antibiotics

Abbreviations: KWM, K‐wire(s) migration; NSAIDs, nonsteroidal anti‐inflammatory drugs; TCS, transcondylar screw; TCSM, transcondylar screw migration.

#### 3.2.1 Association between fracture type, fixation method, major complication occurrence and migration of the TCS

Lateral humeral condylar fractures were associated with a major complication rate of 42.9% (12/28), compared with 1/7 (14.3%) MCHF and BCF which suffered no (0/9) major complications (*p* = .022) (Table 4).

**TABLE 4:** Summary of the complication rates for each fracture type

Fracture type	Number of complications by category (%)					
	None	Minor	Major			Total
			Medical	Surgical	Total	
LHCF	12 (42.9)	4 (14.3)	3	9	12 (42.9)	28 (100)
MHCF	5 (71.4)	1 (14.3)	0	1	1 (14.3)	7 (100)
BCF	9 (100)	0 (0)	0	0	0 (0)	9 (100)

Abbreviations: BCF, bicondylar fracture; LHCF, lateral humeral condylar fracture; MHCF, medial humeral condylar fracture.

Eleven out of 24 (45.8%) fractures stabilized using a TCS and supracondylar K‐wire(s) encountered a major complication, compared with 2/19 (10.5%) using a TCS and epicondylar plate, and 0/1 for the “other” fixation group (Table 5) (*p* = .025). Seven out of 24 (29.2%) TCS placed alongside K‐wire(s) migrated. This compares to no recorded migration of the screw when placed alongside an epicondylar plate (*p* = .022). Fractures stabilized using a TCS and K‐wire(s) were more likely to have any type of complication (14/24 [58.3%]) compared with fractures stabilized with a plate or single TCS (4/20 [20.0%]) (OR 5.60; 95% CI: 1.43; 21.89; *p* = .01) (Table 6). Major complications were seven times more likely in the TCS and K‐wire(s) group than those with a TCS and plate or TCS alone (OR 7.62; 95% CI: 1.44; 40.33; *p* = .009). Fractures that were stabilized with a TCS and K‐wire(s) were also more commonly associated with migration of the TCS (7/24 [29.2%]) compared to TCS and plate or single TCS (0/20 [0%]) (OR 17.57; 95% CI: 0.94; 330.03; *p* = .011). When comparing the use of one k‐wire versus multiple k‐wires within the TCS and K‐wire(s) group, no association was found between complication occurrence (*p* = 1.00), risk of major complication (*p* = .697), or migration of the TCS (*p* = 1.00). The positioning of the wires when multiple were used led to major complications in 3/6 (50%) when placed as a transcondylar and supracondylar wire, and 1/4 (25%) when placed as crossed supracondylar wires.

**TABLE 5:** Summary of the complication rates and evidence of migration of the TCS for stabilization

Fixation method	Complication category		Migration of TCS	
	No intervention (number [%])	Major complication (number [%])	No (number [%])	Yes (number [%])
TCS + K‐wire(s)	13 (54.2)	11 (45.8)	17 (70.8)	7 (29.2)
TCS + plate	17 (89.5)	2 (10.5)	19 (100)	0 (0)
Other	1 (100)	0 (0)	1 (100)	0 (0)

*Note*: The “other” group represents the single transcondylar screw (*N* = 1).

Abbreviation: TCS, transcondylar screw.

**TABLE 6:** Comparison of complication rates in dogs that had fractures stabilized by using a transcondylar screw and supracondylar K‐wire(s) (*N* = 24) (fixation method 1) and stabilization using a transcondylar screw and epicondylar plate (*N* = 19), or transcondylar screw alone (*N* = 1) (fixation method 2)

Type of complication	Fixation method	Number of dogs	Number (%) with complications	OR	95% CI	*p*‐value
Any complication	1	24	14 (58.3)	5.60	1.43, 21.89	.01
	2	20	4 (20)			
Major complication	1	24	11 (45.8)	7.62	1.44, 40.33	.009
	2	20	2 (10)			
Migration of TCS	1	24	7 (29.2)	17.57	0.94, 330.03	.011
	2	20	0 (0)			

Abbreviations: CI, confidence interval; OR, odds ratio; TCS, transcondylar screw.

#### 3.2.2 Association between sex, age, and weight on major complication occurrence or TCS migration

**FIGURE 4:**
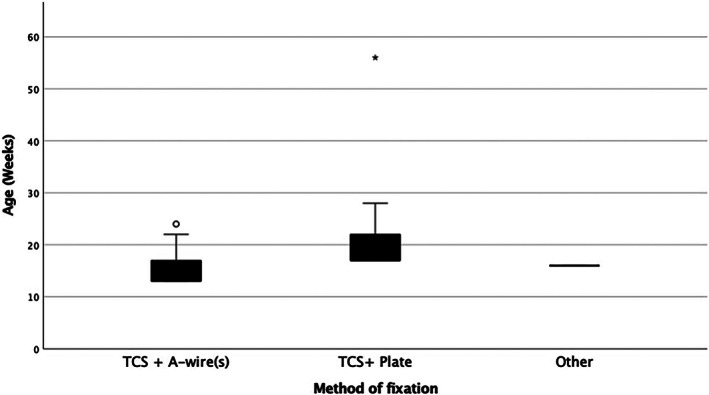
A boxplot showing method of fracture stabilization by age of dog. Transcondylar screw (TCS) and epicondylar plate were more often used in older animals compared to TCS + K‐wire(s) (*p* < .001). The circle and asterisk indicate extreme outliers.

Major complications were associated with younger dogs (*p* = .002), and dogs of lower weight (*p* = .026). Only age was associated with migration of the TCS (*p* = .024), with a lower age more likely to experience migration (Table 7). Older dogs were more likely (*p* < .001) to be stabilized using a TCS+ epicondylar plate than with a TCS and K‐wire(s) (Figure 4).

**TABLE 7:** The effect of signalment, weight, affected leg, and screw placement on complication rates and migration of the TCS

	Complication category			Migration of TCS		
	No intervention	Major complication	*p*‐value	Yes	No	*p*‐value
Age (weeks) (median [range])	17 (13–56)	15 (13–24)	.002	15 (13–17)	17 (13–56)	.024
Sex	17 male, 14 female	6 male, 7 female	.744	2 male, 5 female	21 male, 16 female	.232
Weight (kg) (median [range])	7.1 (3.8–15)	5.2 (4.2–9.4)	.026	5.2 (4.20–8.25)	6.5 (3.80–15.00)	.216
Affected leg	12 left, 19 right	5 left, 8 right	.988	4 left, 3 right	13 left, 24 right	.402
TSA (degrees) (median [range])	6 (0–20)	5 (0–24)	.424	7 (0–24)	6 (0–24)	.730
CW:SD (mean [±SD])	13.9 (±2.2)	14.2 (±2.1)	.651	14.7 (±2.3)	13.9 (±2.1)	.363

Abbreviations: CW:SD, condylar width to screw diameter; TCS, transcondylar screw; TSA, transcondylar screw angle.

#### 3.2.3 Association between age and presence of HIF in contralateral limb

**FIGURE 5:**
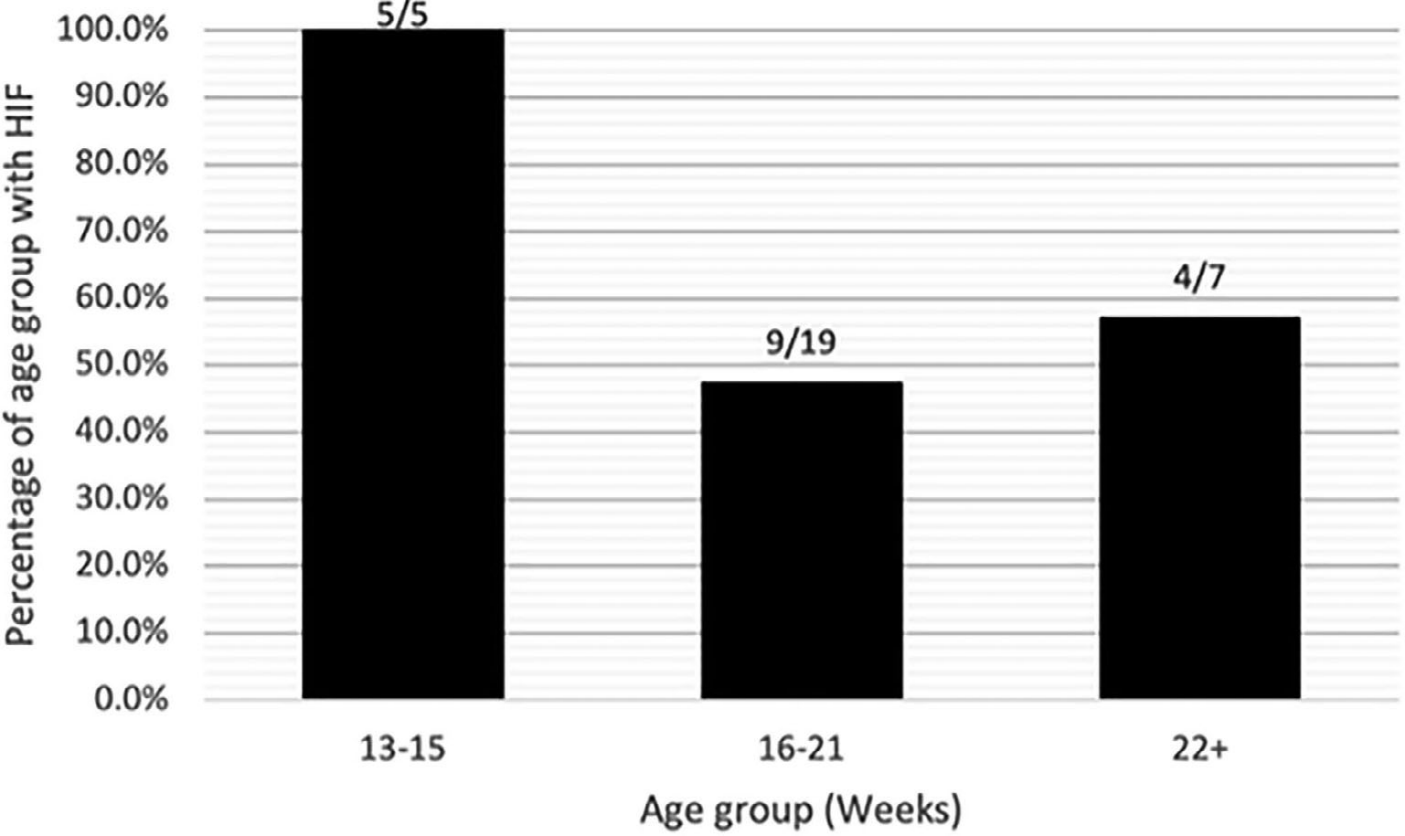
A bar chart showing the percentage of dogs in each age group that had evidence of humeral intracondylar fissure on CT in the contralateral limb (*p* = .129).

**FIGURE 6:**
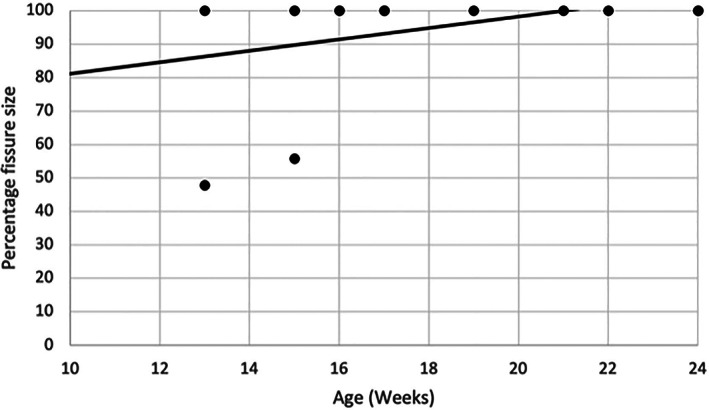
A scatterplot of the percentage fissure length compared to the age of the dog. A moderate positive correlation exists between percentage fissure length and age (*R* = .47, *p* = .048).

Thirty‐one of the 42 dogs included in the study had a CT of the contralateral limb performed at the time of presentation. Of these, 18/31 (58.1%) had evidence of a HIF in the contralateral limb. The presence of a HIF was not more common in younger dogs (*p* = 0.226). Figure 5 illustrates the relationship between the age of patients and the presence of a HIF in the contralateral limb when the dogs are grouped by age. To better characterize fissure size and its relationship with age, the percentage fissure length  was plotted against patient age (Figures 6). A moderate positive correlation (*R* = .47) between age and percentage fissure length was found to be significant (*p* = .048). The correlation between CW:FW and age (*R* = .044) was not significant (*p* = .862).

## 4 DISCUSSION

This study documented the occurrence, risk factors and complication rates of HCF in French bulldogs, alongside the incidence of HIF in the contralateral limb. Fracture morphology distribution was comparable to those previously reported in French bulldogs.^8^, ^30^ LHCF were the most common fracture type, occurring in almost two thirds of HCF, with BCF second and MHCF having the lowest incidence confirming our initial hypothesis. French bulldogs have previously been reported to have a higher rate of MHCF than in other dogs.^8^ While not as high in this study, the rate was higher than the 4%–16% reported in the general dog population.^1^, ^3^, ^5^, ^6^, ^7^, ^8^, ^30^ The reason for this is unknown, but has been noted in other chondrodystrophic breeds. It may be due to the chondrodystrophic conformation of the elbow influencing the loading pattern and therefore fracture type.^8^

In our study, minor and major complications were significantly more common in fractures stabilized with a TCS and K‐wire(s) when compared to those associated with a plate, or when a single TCS was used. Major complications were seven times more likely to occur with a TCS and K‐wire(s) compared to all other stabilization methods. This finding agrees with our hypothesis that HCF stabilization with a TCS and antirotational K‐wire(s) results in a higher complication rate. All the incidences of TCS migration occurred with stabilization with TCS and K‐wire(s). To the authors’ knowledge this is not something specifically previously reported in the literature and is possibly attributed to the stability provided by the different adjunctive fixation modalities in addition to the TCS. The supracondylar K‐wire may not completely neutralize the rotational and shear forces that the fractured condyle must withstand during the healing period, whereas adjunctive plate fixation is biomechanically superior to supplemental K‐wire, and provides increased stiffness.^34^ This should reduce cyclic stress on the screw which can often lead to implant failure.^26^ BCF may therefore have not suffered complications in this study due to all but one case being stabilized with adjunctive plates.

The increased major complication rate seen with TCS and K‐wire(s) may be that HCF in younger dogs were significantly more likely to be stabilized using a TCS and K‐wire(s) compared to other methods. This is probably due to the size of the condyle and the difficulty in applying a plate to these smaller dogs. Dogs of lower age were significantly more likely to experience a major complication and migration of the TCS. This may be attributed to the softer bone stock of younger animals. Significant correlations have been found between pull‐out strength of implants and bone mineral density.^35^, ^36^ Age is possibly a confounding factor for major complications with the K‐wire group, although Perry et al.^24^ found stabilization with TCS and K‐wire(s) was still associated with higher risk of complications, even when age was adjusted for as a confounding factor. Contrary to what would be suspected, a lower weight was also significantly associated with a higher major complication rate, although probably due to lighter dogs also being the youngest.

One HCF was stabilized with a single TCS, that being a MHCF. It would therefore be unwise to make any conclusions about the efficacy and complication rate of this method without a larger sample size. In a study looking at the placement of a single 4.5 mm shaft screw, the single MHCF case in that cohort also did not encounter complications; however, both the LHCF and BCF groups did.^37^ In addition, the placement of a single TCS in the treatment of HIF has previously been associated with high complication rates in some studies.^38^, ^39^ The placement of the screw from medial to lateral as well as some newer repair systems have reported lower complication rates in recent studies.^37^, ^40^

When CT of the contralateral limb was available, 58.1% of dogs had evidence of a HIF. This is similar to that reported in a smaller case series of nine dogs in which 66.7% had evidence of HIF.^31^ In contrast, two larger studies identified no evidence of HIFs.^8^, ^41^ One looked at the contralateral limb in dogs with HCF, the another looked at 74 elbows in nonlame French bulldogs and excluded any dogs younger than 6 months of age. The majority of HCF in this study occurred in skeletally immature dogs, but all still occurred after the ossification centers should have fused, between 8–12 weeks of age.^42^ When looking at the presence of fissures in different aged dogs, this study found that the presence of a fissure did not appear to significantly decrease with age, as would be expected with normal growth.^42^ When assessing fissure size, the fissure width did not decrease with age, but fissure length significantly increased. The 13 dogs that had CT of the contralateral limb available, but did not have evidence of HIF, had a median age of 17 weeks (range 17–22). They were not the older dogs of the cohort whose fissures had fused. These findings reject our hypothesis that the presence of a HIF in the contralateral elbow is due to delayed ossification, and the incidence decreases with age. However, in the experience of the authors, these fractures are seen uncommonly in older dogs which may suggest resolution of the fissure at some stage, though further studies are required. All the dogs studied were under 6 months old, but this suggests that if there is delayed fusion of the humeral condyle ossification centers, then closure is likely to occur later than 6 months of age. IOHC cannot be ruled out and may have a part to play in the increasing prevalence of HCF seen in French bulldogs.

A predisposition to HCF in spaniel breeds has been linked to HIF,^10^ but the pathogenesis in French bulldogs remains unknown. HIF alone can cause ongoing lameness, but also weakens the condyle and therefore predisposes to fracture.^43^ Placement of a TCS across the fissure is advocated to reduce the risk of future fracture.^43^ Placing a screw is not without complications, and so the benefits and risks have to be taken into consideration.^44^ Not every dog with HIF will develop lameness or fracture.^45^ The presence of HIFs found in many of the French bulldogs in this study may indicate that the placement of a TCS is beneficial in this breed if identified on CT, but further studies would be required before this recommendation can be made.

This study was inherently limited by its retrospective nature, the most obvious being that it relied on accuracy of the medical records reviewed and the possible introduction of selection bias. There was no randomization of repair technique or prospective measurements of outcome and it was not possible to standardize surgical preparation, surgical procedure used, and surgical technique. This should be somewhat negated as dogs were from a single referral center, and so similar protocols were used. A further limitation of this study was that long‐term follow‐up was not available for all the dogs included. It is therefore difficult to make conclusions relating to the relatively predictable healing and low incidence of late implant failure in this breed compared with other breeds with documented HIF. In our experience, it is less common to see HCF in adult French bulldogs compared to Spaniel breeds which commonly present with HIF. This may reflect a difference in the underlying etiology and pathogenesis. The classification for reporting and assessing the presence of HIF is prone to error, and although we tried to standardize with a ratio‐based calculation, it is still a subjective measurement. The dogs included were all from a single referral center and so may reflect only local breed and genetic bias. This study was also limited by its small sample size, and so may be potentially underpowered for some associations. In the future, larger studies would be required before conclusions are made. It would also be beneficial to have CT images for older dogs with previously documented fissures to see if the fissures continue to persist with age, and to document whether closure occurs.

There was a significant difference in complication rates between repair methods for the stabilization of HCF in French bulldogs. The use of a TCS and K‐wire(s) has an increased likelihood of major complications than a TCS and plate, and this should be considered when faced with a HCF. We also found that a HIF was present in the contralateral limb of over 50% of the dogs presenting with HCF and may suggest a possible predisposing factor in French bulldogs. Ossification in the humeral condyle of this population of French bulldogs did not appear to occur in the previously described age range of other breeds. Further studies are required to investigate the progression of these fissures.

## CONFLICT OF INTEREST

The authors declare no conflict of interest related to this report.
